# Primary cardiac myxofibrosarcoma: case report, literature review and pooled analysis

**DOI:** 10.1186/s12885-018-4434-2

**Published:** 2018-05-02

**Authors:** Dandan Sun, Yupeng Wu, Yan Liu, Jun Yang

**Affiliations:** 1 Department of Cardiovascular Ultrasound, the First Affiliated Hospital of China Medical University, 155 North Nanjing Street, Heping District, Shenyang, 110001 Liaoning Province China; 2 2nd Department of Neurosurgery, The People’s Hospital of China Medical University and The People’s Hospital of Liaoning Province, Shenyang, 110016 Liaoning Province China

**Keywords:** Primary cardiac mycofibrosarcoma, Clinical features, Follow-up, Prognosis

## Abstract

**Background:**

Primary cardiac myxofibrosarcoma is a very rare cardiac malignancy. The majority of publications are limited to case reports. No pooled analyses of primary cardiac myxofibrosarcoma cases are available. Little clinical features and outcome patterns are acknowledged. The purpose of this study is to identify the clinical characteristics and prognostic factors of primary cardiac myxofibrosarcoma.

**Case presentation:**

A case report of primary cardiac myxofibrosarcoma was presented, and a review of English language literatures of primary cardiac myxofibrosarcomas were performed electronically. Demographics, clinicopathologic data, therapy and follow-up were summarized. The median survival time and the mean survival time were calculated by Kaplan-Meier method. Survival distribution and overall survival were figured by log-rank test and cox proportional hazards models. We present a case, and retrospectively analyzed additional 30 patients derived from 24 isolated articles. The cohort consisted of 18 male and 13 female patients. The age was 41.87 ± 17.89 years. Some common features were found in clinical presentations, pathologic features, treatments and outcome patterns of primary cardiac myxofibrosarcoma. There were special features in echocardiography, histological and immunohistochemical examinations, which should be considered in diagnosis of primary cardiac myxofibrosarcoma. The median survival time/mean survival time (MST) was 14/32.66 months. The median survival time/mean survival time (MST) was 14/32.66 months. Compared to the other groups, the following groups had shorter survival characteristics, including age ≥ 40 years (14/11.79 months), female (14/26.26 months), mass diameter ≥ 40 mm (14/14.64 months), high-grade (2/11.81 months), and no post-treatment (14/28.09 months). Statistical analyses revealed that primary cardiac myxofibrosarcomas were more likely to present with local recurrences and dismal metastases. Tumors ≥ 40 mm in size (*P* = 0.055, HR = 6.79) or with high-grade (*P* = 0.063, HR = 11.45) had significantly worse prognosis.

**Conclusions:**

Primary cardiac myxofibrosarcomas were more likely to present with local recurrences and dismal metastases. Echocardiography, together with histological method should be considered in ordinary diagnosis. Tumors ≥ 40 mm in size or with high-grade had significantly worse prognosis, which should be early diagnosed and treated with rational surgery.

## Background

Primary cardiac tumors are rare, whose incidence varies from 0.3 to 0.7% of all cardiac tumors [1]. Twenty five percent of primary cardiac tumors are malignant. And only 20 % of cardiac neoplasms are primary cardiac sarcomas [2]. And primary cardiac myxofibrosarcoma is even less, which has been only presented in a series of isolated case reports [3–5]. In the World Health Organization classifications of cardiac tumors, myxofibrosarcoma was defined as a malignant tumor composed of fibroblasts with variable amounts of intercellular collagen and abundant myxoid stroma [6]. Patients with primary cardiac myxofibrosarcoma are often asymptomatic until local diffusion or distant metastases occur. The presenting symptoms tend to be nonspecific, that can include dyspnea, palpitation, chest pain, and so on [7]. The prognosis of these tumors is poor and is much influenced by the surgical possibilities [8]. As there are still few series describing primary cardiac myxofibrosarcoma, and the majority of publications are limited to case reports, little characterizations are acknowledged. To the best of our knowledge, there are no large population studies or no accumulated knowledge of primary cardiac myxofibrosarcomas to date. Hereby, we presented a case report of primary cardiac myxofibrosarcoma, and retrospectively analyzed additional 30 patients with primary cardiac myxofibrosarcomas derived from 24 isolated articles in an effort to establish definite clinical presentations, pathologic features, treatments and outcome patterns of primary cardiac myxofibrosarcoma and to develop a rationale for diagnosis and prognostication of this disease.

## Case presentation

**Fig. 1:**
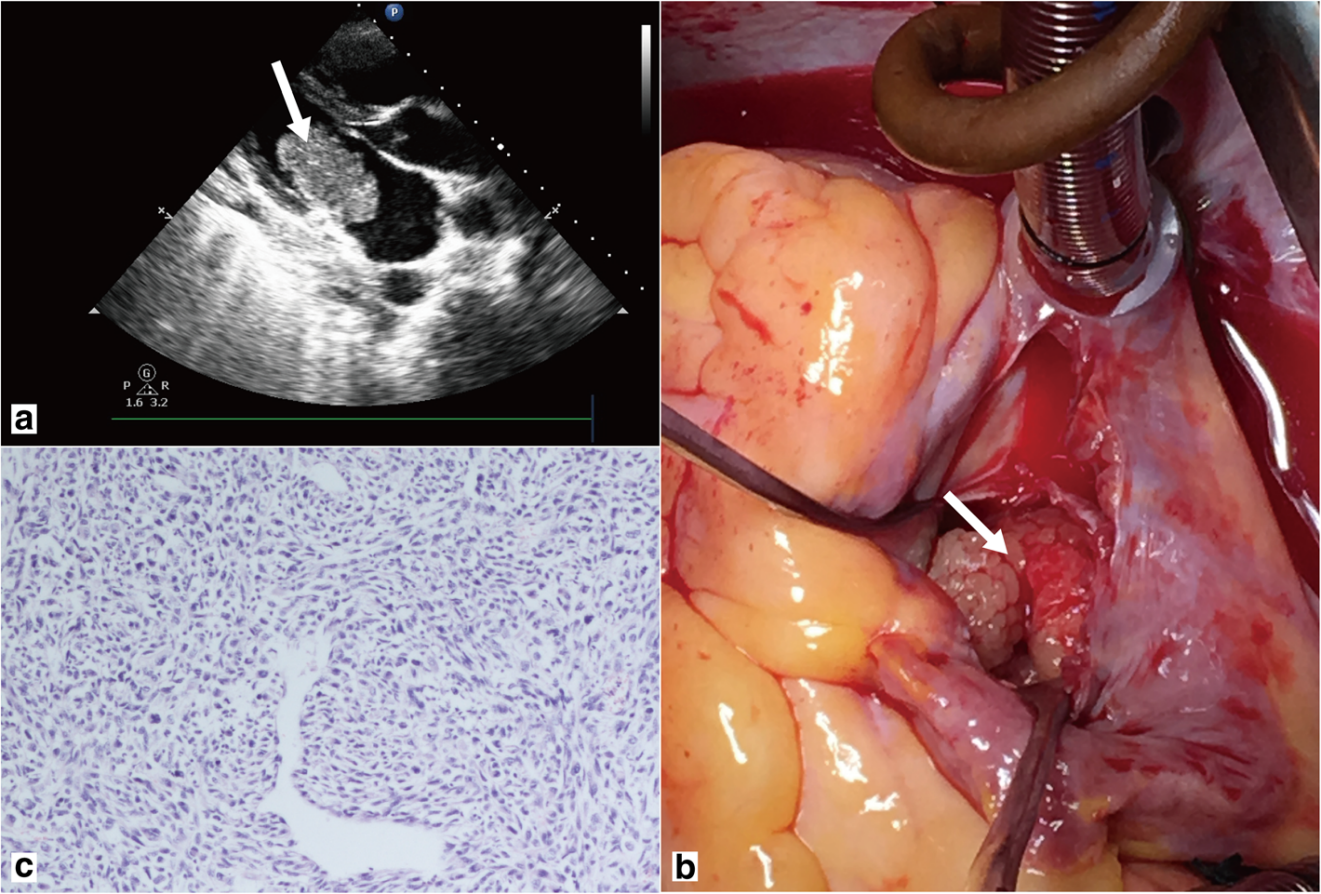
Transthoracic echocardiogram revealed a mass measuring 50 × 35 mm in the left atrium (**a**). The mass was yellow-white with myxoid areas (**b**). Histological examination showed spindle-shaped cells with myxoid background (**c**)

A 34-year-old woman visited our hospital with the complaint of headache for five days, and dyspnea and chest pain for half a month. She had 1-year history of general malaise and poor appetite. The physical examination revealed hypotension, tachycardia with diastolic murmurs. Transthoracic echocardiogram revealed an isoechoic mass measuring 50 × 35 mm in the left atrium. The tumor was attached to the posterior mitral annulus and prolapsed into the left ventricular inflow tract during diastolic phase (Fig. 1a). There was moderate mitral stenosis accompanied with mild regurgitation. The pulmonary arterial systolic pressure was measured as 57 mmHg. Head CT showed an extra-axial lesion in the right parietal-occipital lobe. Cardiac malignant tumor with brain metastasis was suspected. Tumor excision and mitral annuloplasty were performed (Fig. 1b). Traditional histological examination showed that the tumor was composed of spindle-shaped cells with a predominantly myxoid background (Fig. 1c). The tumor cells had anisomorphic and hyperchromatic nuclei. Immunohistochemical test revealed positive immunoreactivity for vimentin, wilms tumor protein 1 (WT1), cluster of differentiation 34 (CD34), cytokeratin, and patchy positivity for myogenin, S-100 protein, CD68, D2–40, and Ki67. It was negative for smooth muscle actin (SMA), desmin, cytokeratin, and epithelial membrane antigen (EMA). Primary cardiac myxofibrosarcoma was diagnosed. Selective operation of brain tumor was advised, which was refused by the patient. And the patient was lost to follow-up.

## Study identification and statistical analysis

**Fig. 2:**
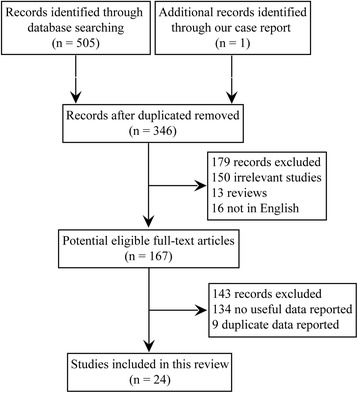
Flow diagram of identification of studies

An extensive review of the English language literatures were performed using the databases of PubMed, Medline, and Web of Science, with keywords of “myxofibrosarcoma”, or “myxoid variant of malignant fibrous histiocytoma”, and “cardiac”, or “heart”. In order to expand the study size, we also included relevent articles obtained from searching references cited by the primary reports. The inclusion critera in the study are as follow: Firstly, originated from the heart chambers, myocardium, or pericardium; Secondly, excluded metastatic cardiac myxofibrosarcoma; Thirdly, identified in the English language literature. Data were collected, including sex, age, clinical presentations, echocardiography findings, pathological features, tumor locations, post-treatments, and follow-up (no evidence of disease, local recurrence, and metastasis) (Fig. 2).

All statistical analyses were performed using the SPSS 16.0 software package (SPSS, Chicago, IL, USA). Discrete variables were expressed as frequencies and percentages, and continuous variables as means ± standard deviations (SD). The Kaplan-Meier method was applied to calculate the median survival time and the mean survival time. The log-rank test was used to test for the survival distributions. Univariate and multivariate Cox proportional hazards models were performed to calculate crude or adjusted hazards ratios (HRs) and 95% confidence intervals (CIs) of overall survival (OS) by different groups with or without adjustment for age and sex. Two-tailed *P* values < 0.05 were considered statistically significant.

## Results

### Clinical features

This study contained 31 well-described cases of primary cardiac myxofibrosarcoma (including our case) in 24 English language literatures [3–5, 7–27]. There were 17 cases from Asian region, 10 cases from American region, and four cases from European region. The cohort consisted of 18 male and 13 female patients. The age at presentation was 41.87 ± 17.89 years (range, 6–90 years).

Presenting complaints were recorded for 28 patients, which were gathered and studied carefully. Finally, three categories were summarized: cardiopulmonary, extra-cardiopulmonary, and nonspecific systemic symptoms (Table 1). The most common cardiopulmonary symptom was dyspnea, accounting for 64.3%, while the most common extra-cardiopulmonary symptom was syncope, representing 21.4%. Physical signs were available in 13 patients, which could be classified as follow: 1. Heart failure, including generalized edema, jugular distension, hepatomegaly, tachypnea, coarse breathing sounds, crackles and rales, hypoxemia, and cyanotic face; 2. cardiac murmurs: II/III diastolic murmur, III/IV pansystolic murmur, and tumor plop; 3. abnormal pulse and blood pressure: hypotension and tachycardia.

**Table 1:** Clinical presentations of patients with primary cardiac myxofibrosarcoma

Variables	N (%)
Cardiopulmonary symptoms	
Dyspnea	18 (64.3%)
Chest pain	9 (32.1%)
Edema	6 (21.4%)
Palpitation	5 (17.9%)
Hemoptysis	3 (10.7%)
Cough	1 (3.6%)
Extra-cardiopulmonary symptoms	
Syncope	6 (21.4%)
Abdominal pain	1 (3.6%)
Headache	1 (3.6%)
Decubitus	1 (3.6%)
Nonspecific systemic symptoms	
Fatigue	2 (7.1%)
Anorexia	1 (3.6%)

### Electrocardiogram feature

Electrocardiograms were provided in 8 patients. Among them, four patients manifested with right axis deviation or right bundle branch block, whose tumors all located in the right ventricle, right atrium, or main pulmonary artery. One electrocardiogram revealed ST segment elevation, indicating possible ST-elevation myocardial infarction. The other three electrocardiograms were normal.

### Imaging findings

Chest x-ray features were presented in 10 patients. Some secondary signs could be seen in these roentgenograms, including enlarged cardiac silhouette, pleural effusion, and pulmonary vascular congestion.

Transthoracic or transesophageal echocardiography was recommended as the first choice of examination. Echocardiography could detect the presence of the intracardiac tumor mass sensitively and conveniently. It could well reflect the location, attachment, and diameter of tumor. Hereby, we would evaluate these aspects from the recordings of echocardiography and intraoperation (Table 2).

**Table 2:** Location, attachment, and diameter of primary cardiac myxofibrosarcoma

Variables	Measurements
Location (*n* = 31)	
Left atrium	18 (58.1%)
Left atrium+pulmonary vein	5 (16.1%)
Right ventricle/right atrium/pulmonary artery	5 (16.1%)
Left ventricle	3 (9.7%)
Attachment (*n* = 20)	
Wall of left atrium/interatrial septum	9 (45.0%)
Entry of pulmonary vein	4 (20.0%)
Mitral valve	3 (15.0%)
Wall of right ventricle/interventricular septum	3 (15.0%)
Tricuspid valve	1 (5.0%)
Mean diameter (mm) (*n* = 22)	43.12 ± 16.18 (12–80)

According to the literatures, left atrium was the most common location, affecting 18 patients (58.1%). The second most common location was left atrium and pulmonary vein, occurred in five patients (16.1%). Other locations included right heart system (involving in right ventricle, right atrium, and pulmonary artery) in five (16.1%), and left ventricle in three (9.7%).

The attachments of tumors were mentioned in 20 patients, which ranged from wall of left atrium to tricuspid valve. In our study, nine tumors attached to the wall of left atrium and left atrial side of interatrial septum (45.0%), and four to the entry of pulmonary vein. Documented attachments included mitral valve in three (15.0%), wall of right ventricle and right ventricular side of interventricular septum in three (15.0%), and tricuspid valve in one (5.0%).

Accurate tumor diameters were reported in 22 patients, ranging from 12 to 80 mm; the mean diameter was 43.12 ± 16.18 mm. The mean diameter of mass in 13 patients was 40 mm or more, and in nine was less than 40 mm.

### Pathological characteristics

Detailed pathologic descriptions were available in 21 literatures. All available pathological descriptions and images were carefully reviewed.

On gross pathologic appearance, the color of mass was often whitish, yellowish, or grayish, sometimes, focal dark-red because of partial hemorrhage. And the shape of mass appeared as nodular, polypoid, papillary, bilobed, or multilobed. The mass often appeared solid in consistency, and most showed myxomatous.

Traditional histological examination revealed some common features. In overall view, the background of tumors was myxoid, which was usually separated with curvilinear thin-walled blood vessels. Some of the tumors had focal hypocellular, to intermediate cellular, or hypercellular area. Most tumor cells exhibited spindle-shaped, occasionally round, polygonal, pleomorphic, or stellate-shaped. A few tumors contained some bizarre giant cells. The cytoplasm was usually atypical, elongated, and eosinophilic. The nuclei were often hyperchromatic and pleomorphic. Most tumor cells had mitotic figures, and some had focal necrosis.

Immunohistochemistry played a key role in the diagnosis of primary cardiac myxofibrosarcoma. The staining properties of various antibodies were summarized in Table 3. Usually, most cases expressed positive in vimentin. Some expressed positive in CD34, SMA, CD117, calponin, Ki67, etc. There were always negative in cytokeratin, desmin, CD31, EMA, S-100 protein, and so on.

**Table 3:** Immunohistochemistrical features of primary cardiac myxofibrosarcoma

Variables	Negative	Positive	
		Focal	Diffuse
CD34	2/9	3/9	4/9
SMA	2/7	4/7	2/7
Ki67	1/5	3/5	1/5
Vimentin	0	3/6	3/6
Calretinin	2/3	0	1/3
S-100 protein	5/6	1/6	0
CD68, myogenin	1/2	1/2	0
PAS	0	0	2/2
CD117, calponin, MDM2, mucicarmin, NSE, WT1	0	0	1/1
D2–40, mucin	0	1/1	0
Cytokeratin, desmin	6/6	0	0
CD31, EMA	3/3	0	0
Caldesmon, factor VIII, MyoD1	1/1	0	0

*CD* cluster of differentiation, *SMA* smooth muscle actin, *PAS* periodic acid schiff, *MDM2* malignant pleural mesothelioma, *NSE* neuron-specific enolase, *WT1* wilms tumor protein, *EMA* epithelial membrane antigen, *MyoD1* myogenic regulatory factor

### Treatments

Overall, 26 patients underwent surgery (83.9%), and five patients did not receive tumor resection (16.1%). Of these 26 patients, 11 were documented with detailed post-treatment, 10 were documented without post-treatment, and 5 were not mentioned. After initial surgery, five patients (23.8%) received chemotherapy; and four (19.1%) received radiotherapy. Two patients (9.5%) were treated with chemoradiotherapy (Table 4). A variety of chemotherapy regimens were recorded, including anthracycline, imatinib, adriamycin, isophosphamide, dacarbazine, cisplatin, etoposide, vincristine, cyclophosphamid, and dactinomycine. The dose of radiotherapy ranged from 40 Gy to 55.8 Gy.

**Table 4:** Details of treatment, post-treatment and follow-up of patients with primary cardiac myxofibrosarcoma

Varibles	N (%)
Treatment (*n* = 31)	
Tumor resection	26 (83.9%)
No tumor resection	5 (16.1%)
Post-treatment (*n* = 21)	
Chemotherapy	5 (23.8%)
Radiotherapy	4 (19.1%)
Chemoradiotherapy	2 (9.5%)
No post-treatment	10 (47.6%)
Follow-up (*n* = 21)	
Local reccurence	9 (42.9%)
Metastasis	4 (19.0%)
NED	8 (38.1%)

*NED* no evidence of disease

### Follow-up and survival analysis

Follow-up data were available for 21 patients. There was a wide range in length of follow-up among the reported case series. Local recurrence and distant metastasis after the initial surgery were the two major adverse events. Of these 21 patients, 13 were documented with adverse event (nine, local reccurence; four, metastasis), and eight had no evidence of disease (NED) (Table 4).

Survival time was calculated using the postoperative time. The median survival time/mean survival time (MST) was 14/32.66 months. Compared to the other groups, the following groups had shorter survival characteristics, including age ≥ 40 years (14/11.79 months), female (14/26.26 months), mass diameter ≥ 40 mm (14/14.64 months), high-grade myxofibrosarcoma (2/11.81 months), and no post-treatment (14/28.09 months) (Table 5).

**Table 5:** The median survival time and the mean survival time calculated by Kaplan-Meier method

Variables	Group	N (%)	Event (N)	Median survival time(m)	Mean survival time(m)
All		21 (100)	13	14	32.66
Age (year)	< 40	12 (57.1)	7	24	43.75
	≥ 40	9 (42.9)	6	14	11.79
Sex	male	11 (52.4)	7	24	30.59
	female	10 (47.6)	6	14	26.26
Location	left heart	18 (85.7)	12	14	25.38
	right heart	3 (14.3)	1	–	49.00
Mean diameter (mm)	< 40	6 (33.3)	1	–	91.20
	≥ 40	11 (66.7)	9	14	14.64
Histologic grading	intermediate/low-grade	5 (55.6)	1	–	68.00
	high-grade	4 (44.4)	4	2	11.81
Post-treatment	yes	11 (52.4)	6	24	28.35
	no	10 (47.6)	7	14	28.09

**Fig. 3:**
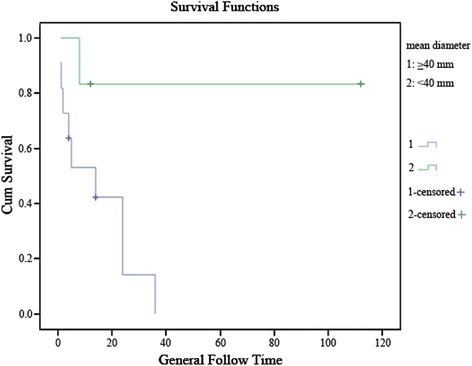
Overall survival comparison between patients with tumor size ≥ 40 mm versus < 40 mm. Months of clinical follow-up are plotted on the x-axes and percentage of surviving cases plotted on the y-axes

**Fig. 4:**
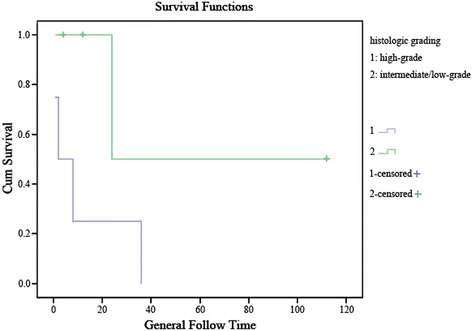
Overall survival comparison between patients with high-grade tumors versus intermediate/low-grade tumors. Months of clinical follow-up are plotted on the x-axes and percentage of surviving cases plotted on the y-axes

Log-rank test and cox proportional hazards models were performed to analyze the risk factors related to the overall survival. There was no significant relationship among age, sex, location, or post-treatment at the overall survival (*P* = 0.220, *P* = 0.725, *P* = 0.299, and *P* = 0.723, respectively). However, the difference in overall survival between mean diameter of tumors ≥40 mm and < 40 mm approached statistical significance (*P* = 0.055, HR = 6.79) (Fig. 3), as well as high-grade tumors and intermediate/low-grade tumors (*P* = 0.063, HR = 11.45) (Table 6) (Fig. 4).

**Table 6:** Overall survival calculated by cox proportional hazards models

Variables	Group	Log-rank *P*-value	Univariate		Multivariate^a^	
			*P-value*	HR (95% CI)	*P-value*	HR (95% CI)
Age (year)	< 40			1 (Ref)		1 (Ref)
	≥ 40	0.220	0.236	2.00 (0.64–6.32)	0.256	1.99 (0.61–6.53)
Sex	female			1 (Ref)		1 (Ref)
	male	0.725	0.730	0.83 (0.28–2.47)	0.968	0.98 (0.31–3.06)
Location	right heart			1 (Ref)		1 (Ref)
	left heart	0.299	0.329	2.77 (0.36–21.51)	0.453	2.35 (0.43–19.10)
Mean diameter (mm)	< 40			1 (Ref)		1 (Ref)
	≥ 40	0.055	0.085	6.38 (0.76–53.29)	0.059	6.79 (0.89–61.42)
Histologic grading	intermediate/low-grade			1 (Ref)		1 (Ref)
	high-grade	0.063	0.103	6.25 (0.69–56.59)	0.081	11.45 (0.74–177.12)
Post-treatment	yes			1 (Ref)		1 (Ref)
	no	0.723	0.729	1.22 (0.40–3.71)	0.668	1.34 (0.54–3.89)

*HR* hazard ratio, *CI* confidence interval, *a* results were adjusted by age and sex

## Discussion and conclusions

In the present study, we have reported a case of primary cardiac myxofibrosarcoma, and reviewed another 30 such cases retrieved from 24 isolated reports. As far as we know, this is the first retrospective study and pooled analysis focusing on primary cardiac myxofibrosarcoma to date. The clinical features (clinical presentations, electrocardiogram features, image findings, pathological characteristics and treatments), follow-up, and survival analysis were summarized in order to develop a rationale for diagnosis and prognostication in primary cardiac myxofibrosarcoma.

Our results revealed that primary cardiac myxofibrosarcoma afflicted relatively young patients, with a mean age of 41.87 years, and no sex preference (male: female, 1.38:1), which was consistent with previous studies [28, 29]. The clinical presentations often varied. Like other benign or malignant cardiac tumors, dyspnea was the most common presentation, which might be caused by the obstruction of atrioventricular valve and ventricular outflow tract [30]. Pathophysiologic changes might result in hypotension, tachycardia, and even syncope [16]. Meanwhile, hemodynamic alterations might lead to heart failure signs and cardiac murmurs [12]. Compared to those with benign cardiac tumors, the patients suffered from primary cardiac myxofibrosarcoma were much more likely to have gastrointestinal and systemic symptoms, which were in accordance with previous studies of cardiac malignant tumors [31]. Electrocardiogram revealed axis deviation, bundle-branch block and ST-T alteration in primary cardiac myxofibrosarcomas. The axis deviation always related to the locations of tumors. The bundle-branch block might be due to the invasive extension. The ST-T changes might be caused by myocardial injury and endocardial damage, or coronary artery embolism due to fragments from the friable tumors [4].

The imaging characteristics were summed up. And transthoracic or transesophageal echocardiography was recognized as the first examination method, which could detect the presence of the intracardiac tumor mass sensitively and conveniently [32]. Transthoracic or transesophageal echocardiography could well reflect the location, attachment, and diameter. We found that the mean tumor size was 43 mm. This was consistent with another report that examined cardiac malignancies [33]. Moreover, it was different from myxomas, which often had smaller tumor size [34]. As expected, the most common site was left atrium, although primary cardiac myxofibrosarcomas presented with notable frequency in right ventricle, pulmonary artery, right atrium, and left ventricle. Hereby, we considered that the attachments of tumors were helpful in differentiating primary cardiac myxofibrosarcoma from cardiac myxoma. The attachments of primary cardiac myxofibrosarcomas were various and multiple, including interatrial septum, atrial wall, entry of pulmonary vein, wall of ventricle, interventricular septum, mitral valve, and tricuspid valve [8, 9, 15]. And the attachment of cardiac myxoma was mainly interatrial septum, especially for the left atrial side [35].

In this series, we also identified some common pathological and histological features, which played an important role in distinguishing primary cardiac myxofibrosarcomas from myxomas and other cardiac malignancies. The characteristics of primary cardiac myxofibrosarcoma were myxoid background, curvilinear thin-walled blood vessels separation, fibroblastic stromal cell element, spindle-shaped tumor cells, and hyperchromatic and pleomorphic nuclei, often in mitotic figures. Because of the myxoid matrix and fibroblastic element, primary cardiac myxofibrosarcoma often appeared whitish, yellowish, or grayish in color [36]. Due to aggressive behavior of the tumor, the presence of hemorrhage within the tumor contributed to focal dark-red color [37]. Different from cardiac myxoma, primary cardiac myxofibrosarcoma often showed irregular in shape, such as nodular, polypoid, papillary, bilobed, or multilobed [16, 17, 24]. Immunohistochemical stains were crucial importance in distinguishing primary cardiac myxofibrosarcomas from other spindle-cell malignancies. Specially, vimentin, SMA and CD34 were markers of myoblast cells that had been shown to be positive in the majority of myxofibrosarcoma specimens [38]. Additionally, Ki-67 was helpful for pathological staging and grading of the tumors [39]. Our results revealed that myxofibrosarcomas were negative for cytokeratin and desmin, which helped us exclude a diagnosis of sarcomatoid carcinoma or leiomyosarcoma [40]. In the majority of cases, S-100 protein also showed negative results, thus melanoma and nerve sheath differentiation were ruled out [41].

Surgery was clearly cornerstone treatment for primary cardiac sarcoma with variable outcomes in the literatures. Patients receiving tumor resections experienced longer survival compared to unresectable ones [17]. And complete surgical resection was always the goal, translating into markedly improved patient survival. Abu Saleh et al. showed that the median survival time of patients who had complete resection was 53.5 months compared with 9.5 months for incomplete resection [42]. In the current study, the median and mean survival time in patients treated with tumor resection were 14 months and 32.66 months, respectively. Five patients expired without surgery due to locally advanced or metastatic disease at presentation. Because of its rarity, there were no reports on local recurrence rates and distant metastases rates of primary cardiac myxofibrosarcoma. The current study happens to be available in the wings. According to our results, the local recurrence rate and distant metastasis rate were 42.9 and 19.0%, respectively, highlighting the aggressive biology of this disease and the need for more effective post-treatment strategies [43]. In our study, 11 patients were documented detailed post-treatment after initial surgery (five received chemotherapy, four received radiotherapy, and two received chemoradiotherapy). However, the role of adjuvant chemotherapy and radiation and their impact on prognosis have remained unclear due to sampling size.

Until now, the prognostic significance of patient and tumor factors on primary cardiac myxofibrosarcoma survival is unclear. Our results showed that the median/mean survival time might tend to be shorter in the following group patients: age ≥ 40 year-old group, female group, mean diameter of mass ≥ 40 mm group, high-grade myxofibrosarcoma group, or no post-treatment group. However, we found that only the mean diameter and histologic grading of tumor had critical statistical significance with overall survival. Tumors ≥ 40 mm in diameter seemed to be more aggressive than those < 40 mm. The patients with large tumor (≥ 40 mm) had taken more risk to suffer poor prognosis than those with small tumor (< 40 mm). Early diagnosis of primary cardiac myxofibrosarcoma might improve the overall survive and raise living quality of the patients [44]. According to our results, one of the five patients with low−/intermediate-grade cardiac myxofibrosarcoma occurred dismal metastasis (right femur), whereas all of the four cases with high-grade cardiac myxofibrosarcoma metastasized or returned in the end. When encountered high-grade cardiac myxofibrosarcoma, complete resection was inescapable. Specially, a more balanced surgical approach in achieving optimal preservation and reconstruction of the cardiac anatomy and function needed to be developed, in order to get better overall survival [43].

Our study had several limitations. Complete follow-up data was unavailable for 32% of patients, which precluded statistical analysis on the impact of some confounding factors on survival, such as immunophenotypic markers and histologic features. In addition, because of its rarity, knowledge on the management and prognosis of primary cardiac myxofibrosarcoma had largely been gleaned from case studies. The follow-up time was randomized and in a wide range. Thus, only the median/mean overall survival was taken into account, not 1-year, 2-year or 5-year overall survival.

Primary cardiac myxofibrosarcoma is a malignant tumor of heart. In addition to presenting a case of primary cardiac myxofibrosarcoma attached in the left atrial side of posterior mitral valve, we conducted a PubMed, Medline, and Web of Science review and identified 30 additional cases of primary cardiac myxofibrosarcoma in the English literature. The current study summarized the definite clinical presentations, pathologic features, treatments and outcome patterns of primary cardiac myxofibrosarcoma. Statistical analyses revealed that primary cardiac myxofibrosarcoma were more likely to present with local recurrences and dismal metastases. Tumors ≥ 40 mm in size or with high-grade had significantly worse prognosis.

Our findings may inform clinicians diagnosing, treating and counseling patients with this rare entity. Imaging examination, especially echocardiography, together with histological and immunohistochemical method should be considered in diagnosis of primary cardiac myxofibrosarcoma, specifically those with tumor sizes ≥ 40 mm. In order to improve prognosis, rational surgery strategies should be developed, specifically those with high-grade. Unfortunately, the limitations of our study design forbid us from making stronger recommendations. Future health information technologies may better aggregate and analyze cancer data, building on meta-analyses and preclinical laboratory studies to more fully understand the biology, behavior, and treatment responses in primary cardiac myxofibrosarcoma.

## Abbreviations

CD34: Cluster of differentiation 34
CI: Confidence interval
EMA: Epithelial membrane antigen
HR: Hazards ratio
MST: Median survival time/Mean survival time
NED: No evidence of disease
OS: Overall survival
SD: Standard deviation
SMA: Smooth muscle actin
WT1: Wilms tumor protein 1
